# The molecular basis of the nonprocessive elongation mechanism in levansucrases

**DOI:** 10.1074/jbc.RA120.015853

**Published:** 2020-12-17

**Authors:** Enrique Raga-Carbajal, Adelaida Díaz-Vilchis, Sonia P. Rojas-Trejo, Enrique Rudiño-Piñera, Clarita Olvera

**Affiliations:** 1 Departamento de Ingeniería Celular y Biocatálisis, Instituto de Biotecnología, Universidad Nacional Autónoma de México, Cuernavaca, Morelos, México; 2 Departamento de Medicina Molecular y Bioprocesos, Instituto de Biotecnología, Universidad Nacional Autónoma de México, Cuernavaca, Morelos, México

**Keywords:** Fructan, fructooligosaccharide, enzyme structure, carbohydrate-binding sites, acceptor subsites, enzyme mechanisms, crystal structure, fructansucrases, DP, degree of polymerization, FOS, fructooligosaccharide, HMW, high molecular weight, HPAEC-PAD, high-performance anion-exchange chromatography with pulse amperometric detection, LMW, low molecular weight, LS, levansucrase, MW, molecular weight, OB, oligosaccharide binding, SacB, *Bacillus subtilis* levansucrase

## Abstract

Levansucrases (LSs) synthesize levan, a β2-6-linked fructose polymer, by successively transferring the fructosyl moiety from sucrose to a growing acceptor molecule. Elucidation of the levan polymerization mechanism is important for using LSs in the production of size-defined products for application in the food and pharmaceutical industries. For a deeper understanding of the levan synthesis reaction, we determined the crystallographic structure of *Bacillus subtilis* LS (SacB) in complex with a levan-type fructooligosaccharide and utilized site-directed mutagenesis to identify residues involved in substrate binding. The presence of a levanhexaose molecule in the central catalytic cavity allowed us to identify five substrate-binding subsites (−1, +1, +2, +3, and +4). Mutants affecting residues belonging to the identified acceptor subsites showed similar substrate affinity (*K*m) values to the wildtype (WT) *K*m value but had a lower turnover number and transfructosylation/hydrolysis ratio. Of importance, compared with the WT, the variants progressively yielded smaller-sized low-molecular-weight levans, as the affected subsites that were closer to the catalytic site, but without affecting their ability to synthesized high-molecular-weight levans. Furthermore, an additional oligosaccharide-binding site 20 Å away from the catalytic pocket was identified, and its potential participation in the elongation mechanism is discussed. Our results clarify, for the first time, the interaction of the enzyme with an acceptor/product oligosaccharide and elucidate the molecular basis of the nonprocessive levan elongation mechanism of LSs.

Levans are carbohydrates such as β2-6 fructose oligo- and polysaccharides that have broad applications as prebiotic, anticancer, antibacterial, antioxidant, anti-inflammatory, and antiobesity agents (1, 2, 3, 4, 5, 6, 7). Owing to this wide diversity of applications, the development of methods by which to produce these relevant saccharides is of great interest for several industrial sectors, such as the food, cosmetics, and pharmaceutical industries.

In nature, levans are produced from sucrose by levansucrases (LSs), which are enzymes produced primarily by bacteria. LSs (EC 2.4.1.10) catalyze the transfer of the fructosyl moiety from sucrose to an acceptor molecule. In this enzymatic reaction, the sucrose glycosidic linkage is broken, resulting in the formation of a covalent fructosyl-enzyme intermediate and the release of glucose (8). The fructosyl moiety is then transferred from the enzyme to an acceptor molecule, from which two types of reactions can occur. The transfructosylation reaction occurs if the acceptor is sucrose, fructose, glucose, or a growing levan chain. However, if the acceptor is a water molecule, the enzyme releases fructose, completing the sucrose hydrolysis reaction.

Among bacterial LSs, some enzymes preferably produce polymers, whereas other enzymes generate large amounts of small fructooligosaccharides (FOSs) with a consequent lower yield of levan (9). Recently, it was demonstrated that the LS from *Bacillus subtilis* (SacB) is capable of synthesizing polymer distributions with different molecular weights through two distinct elongation mechanisms (10). A high-molecular-weight (HMW, >2000 kDa) levan is produced through a processive mechanism, which consists of successive incorporations of fructose units into a single growing chain retained by the enzyme until the elongation process is finished. On the other hand, a low-molecular-weight (LMW, 7.2 kDa) levan is synthesized by employing a nonprocessive mechanism, in which the fructose units are added to growing chains but are taken up and released into the solution according to their affinity for the enzyme (10). These observations indicate that SacB has the structural machinery for both elongation mechanisms. At present, the crystallographic structures of LSs from *B. subtilis, Gluconacetobacter diazotrophicus*, *Bacillus megaterium*, *Erwinia amylovora*, and *Erwinia tasmaniensis* have been determined (8, 11, 12, 13, 14). All of these structures display a single domain with a five-bladed β-propeller architecture, in which each β sheet adopts the classical “W” topology of four antiparallel β strands. The β-propeller structure encloses a funnel-like central cavity that is negatively charged at the site where the substrate binds. At the bottom of the cavity, where the catalytic triad is located, there are two aspartic acids and one glutamic acid. In SacB, residues D86 and E342 have been identified as the nucleophile and the general acid/base, respectively, whereas D247 is the transition state stabilizer (8).

Catalytically inactive mutants of SacB have been crystallized in complexes with donor substrates, namely, sucrose and raffinose (8, 15). These substrates were located at the active site, revealing the amino acids constituting the substrate-binding subsites (−1, +1, and +2). The −1 subsite contains residues, namely, W85, D86, W163, R246, and D247, in contact with the fructosyl unit from sucrose. Similarly, the residues in contact with the glucosyl unit that comprise the +1 subsite are R246, E340, E342, and R360 (8). Furthermore, residues N242 and Y237 were identified as part of the +2 subsite, as they are responsible for only a few water-mediated interactions with the galactosyl unit from raffinose (15). Site-directed mutagenesis studies on LSs have revealed that modifications of residues N242, K363, and Y237 (numbering of *B. subtilis* SacB), which are located on the surface of the catalytic cavity, affect the enzyme catalytic efficiency, transfructosylation, and hydrolysis ratio by interrupting the polymerization process, and thus, these residues control the chain lengths of levans (12, 16, 17, 18, 19). However, the interactions mediated by these residues have not yet been identified, and the existence of additional external acceptor-binding subsites acting as structural determinants in the elongation of levans has not yet been clarified. Thus, it is necessary to study the interactions of the enzymes with intermediate products to identify the levan recognition sites involved in polymerization. This information will potentially enable us to rationally modify these enzymes to generate custom products with size-defined properties.

In this work, we present the crystal structure of an inactive double-site mutant of SacB (D86A/E342A) in complex with levan-type hexasaccharides, which are intermediate products in the synthesis of LMW levans from sucrose (20). The interactions of the residues belonging to the binding subsites (−1, +1, and +2) with this natural acceptor were clarified, and external subsites (+3 and +4) identified. We also investigated the role of residues belonging to the acceptor subsites in the levan elongation mechanism by evaluating the effect of their mutation on the kinetic properties and product specificity of the enzyme. In addition, a new oligosaccharide-binding (OB) site (oligosaccharide binding site 2, OB2 site) in the vicinity of the catalytic pocket has been revealed, and its potential involvement in the polymerization reaction described.

## Results

### The crystal structure of the SacB-oligosaccharide complex shows two OB sites

**Figure 1:**
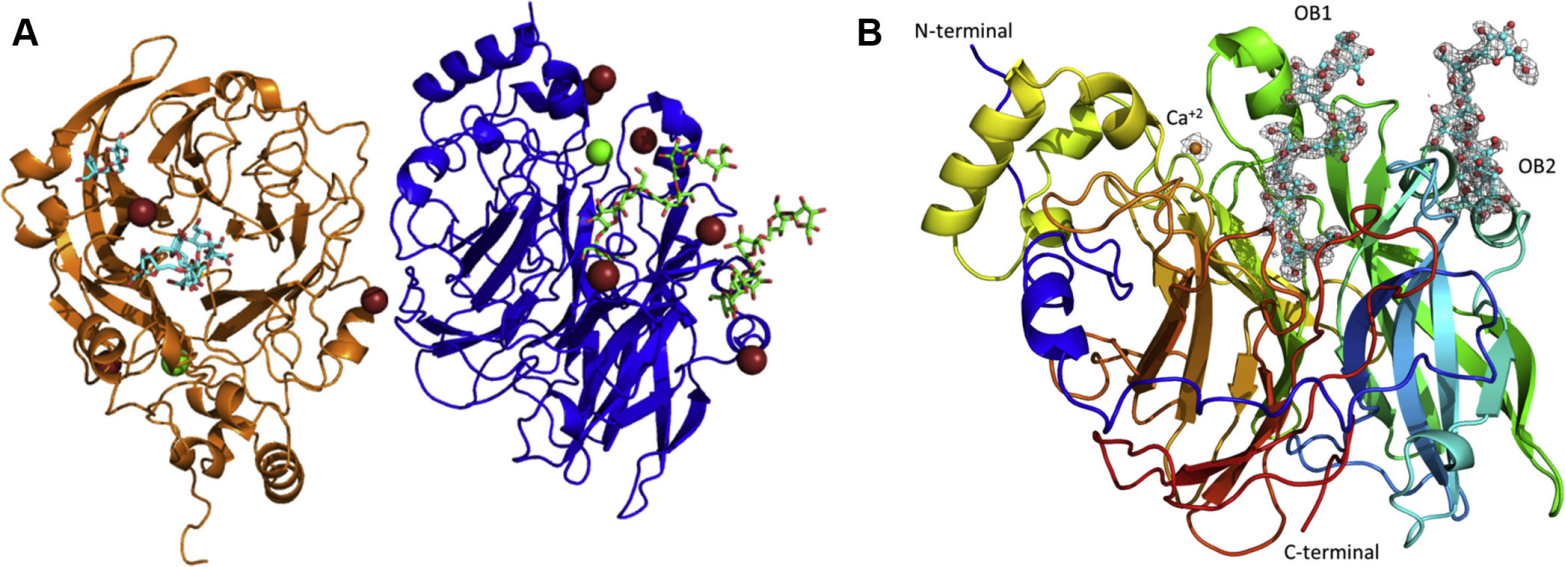
**Crystal structure of the SacB-oligosaccharide complex.***A*, chain A (*blue*) and chain B (*orange*) are present in the crystal asymmetric unit. *Green* and *red spheres* correspond to calcium and bromide ions, respectively. Oligosaccharides are represented as *sticks*, with carbon atoms in *green*, chains A and B in *cyan*, and oxygen molecules in *red*. *B*, the overall structure of chain A with an active site containing six fructose units of the bound levanhexaose molecule (OB1 site) and six other fructose units 20 Å away from the active site (OB2 site). Each oligosaccharide has the corresponding omitted electron density map contoured at the 1.0 σ level.

The structure of an inactive double mutant of SacB (D86A/E342A) cocrystallized in the presence of a levan-type FOS 6,6,6,6,6,6-kestooctaose (with a degree of polymerization [DP] of 8) was determined (Protein Data Bank [PDB] ID: 6VHQ) by molecular replacement using the available coordinates of wildtype (WT) LS from *B. subtilis* (PDB ID: 1OYG) (8) and refined to a final R of 16.7% (R_free_ of 23.4%) at a 2.05 Å resolution (Table S1). The crystal asymmetric unit contains two molecules of SacB, chains A and B, each including residues 32 to 471 (Fig. 1*A*). The root-mean-square deviation (RMSD) between both chains using 440 Cα atoms is 0.26 Å. This crystal structure is practically identical to that of WT SacB (PDB ID: 1OYG), with an RMSD of 0.35 Å using 438 Cα atoms. Each chain contains a hepta-coordinated Ca^2+^ ion, water molecules, and bromide ions in different sites, which are from the crystallization condition and two oligosaccharide molecules. Although the enzyme was cocrystallized with an octasaccharide, only the electronic densities of six fructose units bound by β2-6 glycosidic bonds were observable at the active site of both SacB molecules (oligosaccharide binding site 1, OB1 site). There was no electronic density for the fructose and glucose units remaining in the kestooctaose molecule, perhaps because of the high flexibility of these units, which were exposed to the solvent. Hereafter, this ligand is referred to as levanhexaose. Furthermore, the electronic density for a second oligosaccharide was located approximately 20 Å from the catalytic pocket (oligosaccharide binding site 2, OB2 site). The additional electronic densities were modeled with levanhexaose and levanbiose (difructose) in chains A and B, respectively, and both are fructose chains with β2-6 glycosidic bonds. The SacB-oligosaccharide crystal structure is shown in Figure 1*B*.

Superimposition of SacB (D86A/E342A) (PDB ID: 6VHQ, chain A) with the coordinates of LSs from *G. diazotrophicus* (PDB ID: 1W18), *E. amylovora* (PDB ID: 4D47), and *E. tasmaniensis* (PDB ID: 6FRW) gave an RMSD of 1.7 Å using approximately 345 Cα atoms, and superimposition of SacB (D86A/E342A) with *B. megaterium* LS N252A (PDB ID: 3OM5) gave an RMSD of 0.62 Å using 429 Cα atoms, indicating a higher structural similarity between LSs from the *Bacillus* genus.

### OB1 site

**Figure 2:**
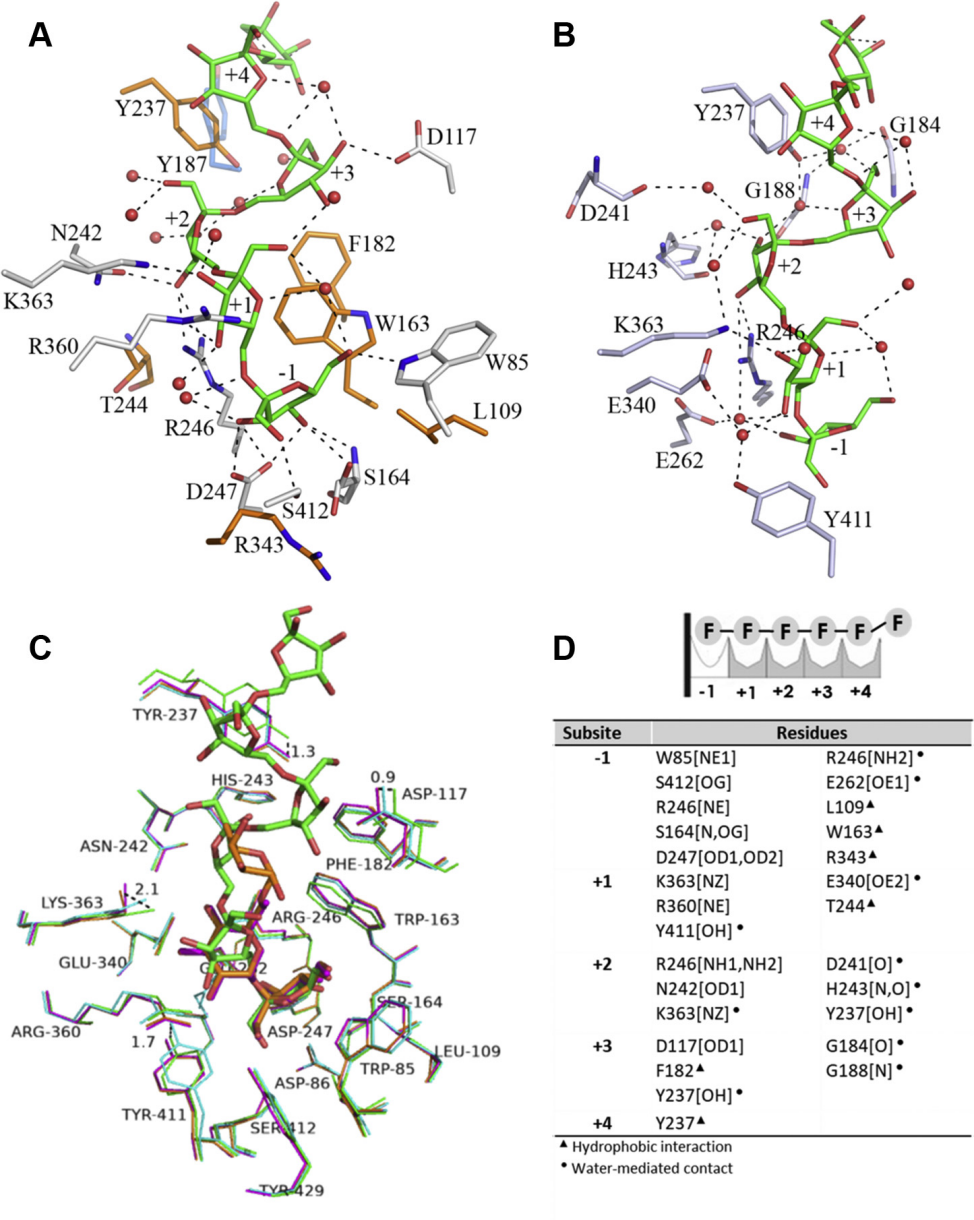
**OB1 site in the SacB-levanhexaose complex.***A*, direct residue–ligand interactions. The six fructose units of the levanhexaose molecule are shown in *green*. Hydrogen bonds involving protein residues and water molecules (*red spheres*) are shown as *black dashed lines*. The *orange residues* interact through hydrophobic interactions. *B*, water-mediated residue–ligand interactions. *C*, view of the active sites of complexes SacB-levanhexaose (*green*, PDB ID: 6VHQ), SacB-sucrose (*magenta*, PDB ID: 1PT2), SacB-raffinose (*orange*, PDB ID: 3BYN), and SacB (apo form, *cyan*, PDB ID: 1OYG) superimposed. *Dashed lines* indicate the distances between different conformations that a residue adopts in complex with different ligands. *D*, summary of the residues belonging to the five subsites identified in the OB1 site. Atoms that make contact with the carbohydrate are indicated in brackets. The following symbols represent the contact type: hydrophobic interactions are shown as *solid triangles*, *solid dots* represent interactions mediated by water, and hydrogen bonds have no symbol.

As already mentioned, inside the negatively charged central pocket of both SacB (D86A/E342A) chains, six fructose units bound by β2-6 glycosidic bonds of the octasaccharide molecule were modeled with a well-defined electronic density. The levanhexaose molecule is oriented with its nonreducing end at the interior of the active site pocket and its reducing end protruding out of the central pocket. The levanhexaose is bound to the enzyme by 32 hydrogen bonds with the residues in the catalytic pocket, 12 to the side chains, 1 to the backbone (Fig. 2*A*), and the rest of the interactions with 11 residues through water molecules (Fig. 2*B*). CH/π stacking interactions with W163 and Y237 and hydrophobic interactions with L109, F182, G184, Y187, T244, R343, and Y411 also stabilize levanhexaose binding (Fig. 2*A*).

Two crystal structures of SacB have been determined, one in complex with sucrose (PDB ID: 1PT2) and one in complex with raffinose (PDB ID: 3BYN), and both substrates have preferential donor characteristics (8, 15). In addition, the crystal structure of *E. amylovora* LS has been determined with the sucrose hydrolysis products, fructose and glucose, leaving the active site (13). The LS complexes with sucrose and raffinose identified the substrate-binding −1, +1, and +2 subsites, according to the nomenclature of (21). In the SacB-levanhexaose structure, the oligosaccharide is used as a ligand, despite not being a donor substrate, and it is placed with its nonreducing end in an identical orientation to the fructosyl unit of sucrose and raffinose in their respective crystal structures, which allows us to define the −1, +1, +2, +3, and +4 subsites, beginning with the fructose unit inside the active site (−1) and ending with the fructose unit (+4) that extends toward the solvent (Fig. 2).

The SacB-levanhexaose complex shows that the −1 subsite is conserved with respect to the sucrose and raffinose complexes, in which the fructose units of the three oligosaccharides are shown in very similar positions (Fig. 2*C*). This subsite is formed by residues W85, L109, W163, S164, R246, E262, R343, S412, and Y429 and catalytic residues D247, D86, and E342 (Fig. 2*D*).

At the +1 subsite, the levanhexaose fructose ring is rotated approximately 30° in comparison with the sucrose and raffinose glucose rings (Fig. 2*C*). Despite this, it conserves many hydrogen bonds that occur in these substrates, such as those generated by the side chains of E340, R360, K363, and Y411. It should be noted that, in chain A, OB generates displacement of the Y411 and K363 side chains (Fig. 2*C*). In the first case, the Y411 hydroxyl group moves 1.7 Å (∼8 times the coordinate error of PDB ID: 6VHQ) in relation to the apo form, moving away from the ligand but maintaining an interaction with fructosyl 2 through a water molecule located in this place as result of the acid/base residue mutation (E243A). This Y411 movement is also observed in the complexes with sucrose and raffinose, so it must be a common structural adjustment for the binding of any substrate. In the case of K363, a displacement of 2.1 Å (9 times the coordinate error of PDB ID: 6VHQ) was observed regarding the position described in the structures with sucrose and raffinose. Of interest, Y411, R360, and K363 show a different conformation in chain B, with the position of Y411 being similar to that of the apo SacB structure, while R360 and K363 shift their positions by 6.0 and 4.5 Å, respectively, away from their positions in chain A (Fig. S1). The conformations in chain B change the interactions of these residues with fructosyl 2, producing direct interactions with the side chains of Y411 (OH) and R360 (NE and NH1), and no contact with K363 was found. These different states reflect the flexible conformation of these three residues in the catalytic pocket.

At the +2 subsite, substantial differences in the conformation of the crystallized ligands are marked, with clearly differentiated positions. In the raffinose complex, the galactose unit interacts through water-mediated hydrogen bonds with residues Y237, N242, and R246 and the main chain of A116 (15). In the case of the SacB-levanhexaose complex, the fructose ring interacts by two hydrogen bonds with the side chains of N242 and R246 at the fructosyl 3, and it also has hydrogen bonds through water molecules with the side chains of K363 and Y237 and the backbones of H243 and D241 (Fig. 2, *A*–*B*).

The crystallographic structure of the SacB-levanhexaose complex allows us to analyze, for the first time, the existence of higher-binding subsites that participate in the binding of long-chain substrates at the catalytic cavity exit. However, because the ligand is more exposed to the solvent, few interactions are identified (Fig. 2, *A*–*B*). In this way, it can be established that the side chains of D117 and F182 participate in the +3 subsite, the former through a hydrogen bond with fructosyl 4 and the latter through a hydrophobic interaction. It is important to note that F182 is positioned to support a stacking interaction with W163, which is an important residue that coordinates ligand binding in subsites −1 and +1. Additional interactions in subsite +3 are those that occur through the side chain of Y237 and the main chains of G184 and G188 that interact *via* three water-mediated hydrogen bonds. In the next subsite, the +4 subsite, residue Y237 forms CH/π stacking interactions with the fifth fructose unit of the ligand. In this subsite, ligand binding promotes notable movements of residues D117 and Y237, which shift 0.8 and 1.3 Å (∼4 and ∼6 times the coordinate error of PDB ID: 6VHQ) away from their positions in the apo form, respectively (Fig. 2*C*). Finally, no interactions were observed between the enzyme and the sixth fructose unit of the ligand that protrudes from the catalytic pocket; however, 4.9 Å away from the sixth fructose unit, residue Y187 was of interest because its aromatic ring faced the bulk solution in a manner that might support the interaction of more extended ligands.

### A novel OB site in SacB

**Figure 3:**
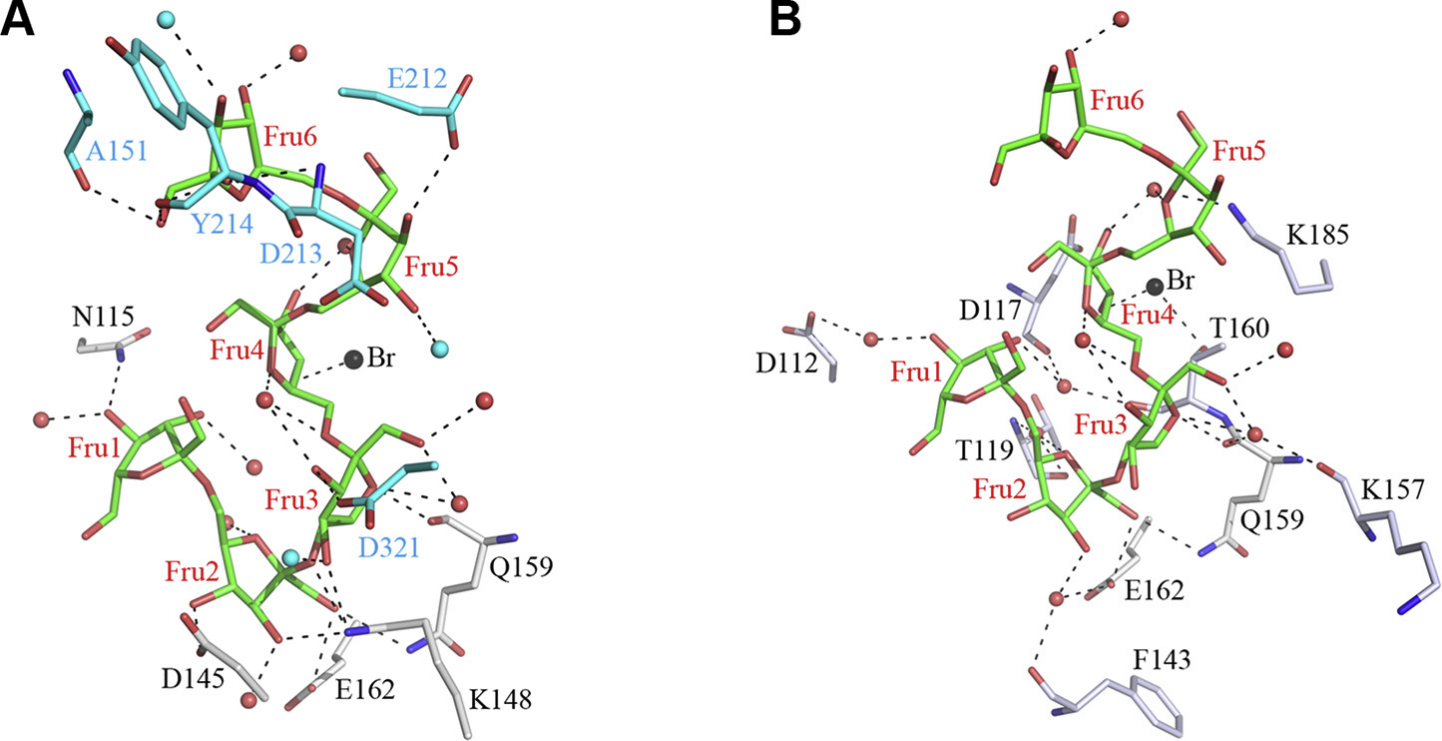
**OB2 site in chain A of the SacB-levanhexaose complex.***A*, direct residue–ligand interactions. The fructose units of the levanhexaose molecule are shown in *green*, and hydrogen bonds are shown as *black dashed lines*. *Red* and *black spheres* correspond to water molecules and bromide ions, respectively. In addition, the residues and water molecules belonging to a neighboring symmetric chain in contact with the levanhexaose molecule are shown in *cyan*. *B*, water-mediated residue–ligand interactions.

Of interest, in addition to the presence of the oligosaccharide at the active site, chain A of the SacB-levanhexaose complex has a second oligosaccharide that was also modeled as a levanhexaose molecule (Fig. 1*A*), revealing a secondary OB site (OB2 site). This external binding site is located on the enzyme surface approximately 20 Å from the bottom of the catalytic cavity. The oligosaccharide is bound to the enzyme by 23 hydrogen bonds; 1 to the backbone of Q159; 7 to the side chains of N115, D145, K148, Q159, and E162; and 15 to water molecules (Fig. 3, *A*–*B*). In addition, the oligosaccharide has a bromide ion bound to it. Furthermore, there are 10 additional hydrogen bonds between the oligosaccharide and the neighboring symmetric chain in the crystal (Fig. 3*A*).

The interaction of the OB2 site is mediated by the aforementioned five residues that directly contact the three fructose units at the nonreducing end of the oligosaccharide (Fig. 3*A*). Fructosyl 1 forms a hydrogen bond with the N115 side chain and three hydrogen bonds through water molecules with the side chain of D112 and the backbones of D117 and T160. Fructosyl 2 has the largest number of hydrogen bonds. It forms five hydrogen bonds with the side chains of D145, K148, Q159, and E162. In addition, fructosyl 2 has two hydrogen bonds through water molecules with the E162 side chain and the main chains of T119 and F143. Here, there are two movements in the side chains of D145 and K148 in comparison with the SacB apo form (Fig. S2), with D145 moving 1.3 Å (∼6 times the coordinate error of PDB ID: 6VHQ) and K148 moving 0.5 Å (∼2 times the coordinate error of PDB ID: 6VHQ). Fructosyl 3 forms two hydrogen bonds with the Q159 main chain and the K148 side chain and five hydrogen bonds with three water molecules. In addition, this fructosyl moiety has two hydrogen bonds through water molecules with the main chains of K157 and Q159, as well as two hydrogen bonds with the D321 side chain and a water molecule of the neighboring symmetric chain. Fructosyl 4 forms two hydrogen bonds with two water molecules and is bound to a bromide ion at a hydrogen-bonding distance of 3 Å. Likewise, fructosyl 4 forms two hydrogen bonds through water molecules with the K185 side chain and through the bromide ion with the T160 side chain. Fructosyl 5 forms two hydrogen bonds, one with E212 and one with a water molecule of the neighboring chain in the crystal. Finally, fructosyl 6 forms six hydrogen bonds with a water molecule and the main chains of A151, D213, and Y214 (Fig. 3*A*).

It is worth noting that, in chain B, only two β2-6 linked fructose molecules (levanbiose moiety) could be modeled on the OB2 site, corresponding to the fructosyl 2 and 3 moieties of the levanhexaose in chain A, which are indeed the moieties with the higher number of contact points with the enzyme. The missing sugar units may have high flexibility owing to exposure to the solvent and the absence of crystal packing contacts, as observed in chain A.

### Mutation of residues involved in the OB1 site

**Figure 4:**
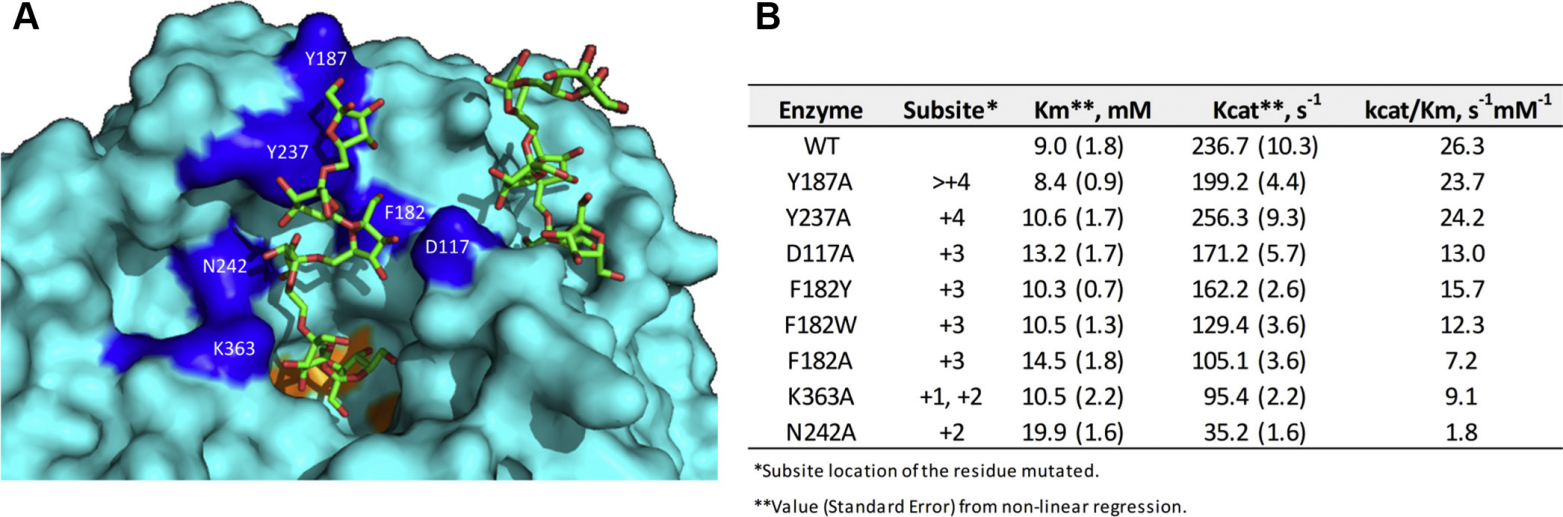
**Position and kinetic properties of SacB mutants.** Location of the mutated residues on the surface of the SacB structure (*A*). Subsite and kinetic parameters of the SacB variants (*B*).

To further study the functional role of the OB1 site in levan elongation, six SacB variants were constructed by individually mutating single amino acids, which are involved in the +2 (N242, K363), +3 (D117, F182), and +4 acceptor subsites (Y237) and beyond (Y187), into an alanine residue (Fig. 4*A*). The alanine mutation was intended to abolish the interactions between the side chains of the residues and the oligosaccharides without affecting the protein backbone. In the case of residue F182, we performed additional mutations to tyrosine and tryptophan to attempt to preserve the stacking interaction observed with residue W163 (EWSG motif), which is an important residue for sucrose binding (22, 23). Eight SacB mutants (N242A, K363A, D117A, F182A, F182Y, F182W, Y237A, and Y187A) were expressed and purified, and their kinetic parameters were determined (Fig. 4*B*). The variants showed Michaelis–Menten constants comparable with that of the WT (9 mM), except for the 1.6- and 2.2-fold increase in the substrate affinity (*K*m) in variants F182A (14.5) and N242A (19.9 mM), respectively. All mutants, excluding Y237A (256.3 s^−1^), showed a decrease in the *k*_cat_ value compared with that of the WT (236.7 s^−1^), ranging from 199.2 to 35.2 s^−1^. Of note, the variants of F182 exhibited *k*_cat_ values reduced by 1.4- to 2.2-fold, following the order Y > W > A. Considering that the kinetic constant can be influenced by slight structural changes besides mutations, in global terms, all the mutations induced a reduction in the enzyme catalytic efficiency (WT, 26.3 s^−1^ mM^−1^), which was correlated with the distance from the active site of the mutated residue.

**Figure 5:**
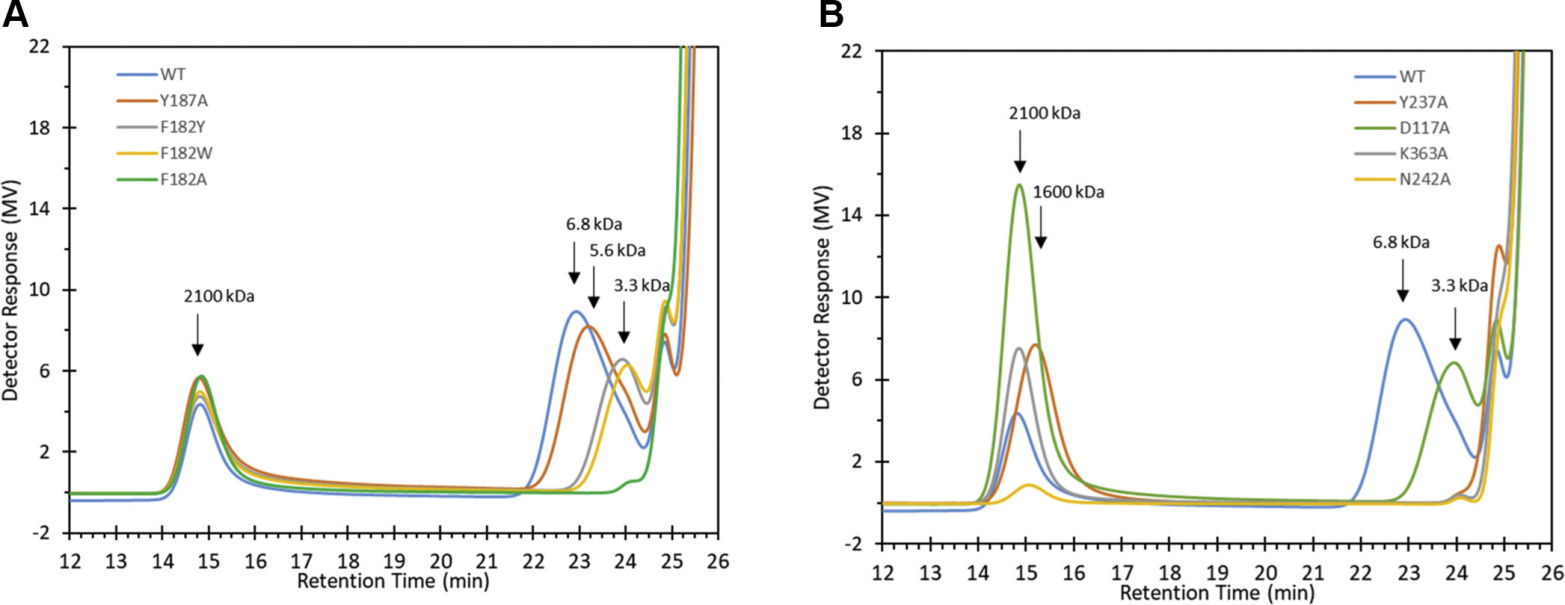
**Gel permeation chromatograms of the products synthesized by SacB and its mutants.** Reaction conditions: 0.1 μM enzyme, 300 mM sucrose, 37 °C, and pH 6. The reactions were analyzed at a sucrose conversion of over 70%.

The product specificity of the variants was evaluated under reaction conditions in which SacB typically synthesizes a bimodal levan molecular weight distribution, *i.e.*, 300 mM sucrose and 0.1 μM (5.5 μg/ml) SacB (10). Gel permeation chromatography analysis showed bimodal product distributions for mutants Y187A, F182W, F182Y and D117A (Fig. 5), preserving the formation of a HMW distribution (MW>2000 kDa) and reducing the length of the LMW products to 5.6 kDa for the case of Y187A and to 3.3 kDa for the other three mutants. In contrast, variants F182A, K363A, and Y237A synthesized only HMW levans. In the case of N242A, HMW levans were barely formed.

**Figure 6:**
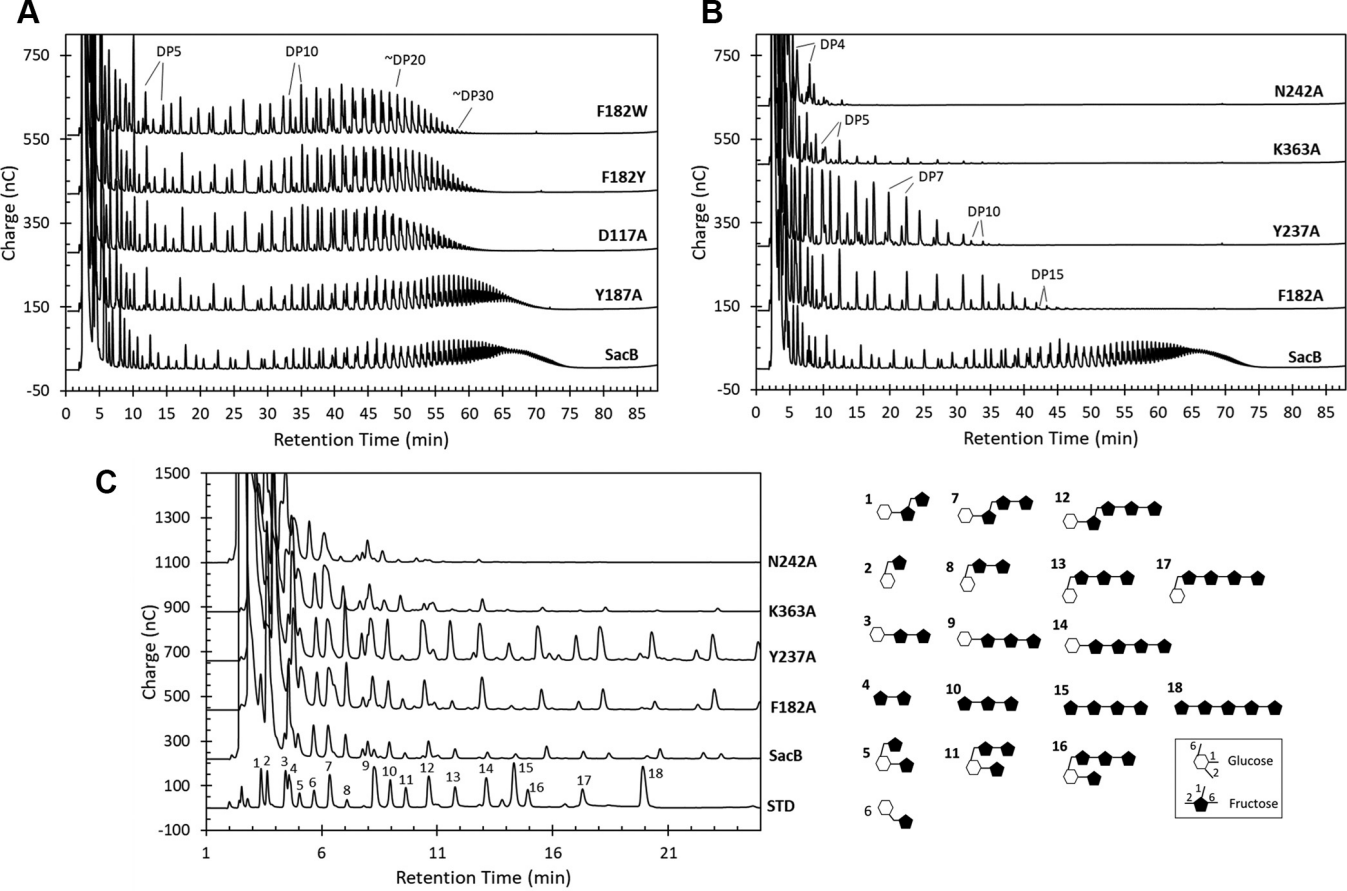
**HPAEC-PAD analysis of the oligosaccharides synthesized by SacB and its mutants**. Reaction conditions: 0.1 μM enzyme, 300 mM sucrose, 37 °C, and pH 6. The reactions were analyzed at a sucrose conversion of over 70% (*A*–*B*). *C*, amplified view of chromatogram (*B*). Oligosaccharide identification (structures included are): 1-kestose (1), blastose (2), 6-kestose (3), levanbiose (4), neo-kestose (5), ercose (6), 1,6-nystose (7), blastotriose (8), 6,6-nystose (9), levantriose (10), 6-neo-nystose (11), 1,6,6-kestopentaose (12), blastotetraose (13), 6,6,6-kestopentaose (14), levantetraose (15), 6G,6,6-kestopentaose (16), blastopentaose (17), and levanpentaose (18).

The high-performance anion-exchange chromatography with pulse amperometric detection (HPAEC-PAD) oligosaccharide profile corroborated the mutation effect on the size delimitation of the LMW products (Fig. 6, *A*–*B*). The mutant Y187A (residue located beyond subsite +4) had a similar profile to that of the WT, with only a slight decrease in the amount of the largest products (65–75 min in Fig. 6*A*). Subsite +3 mutants D117A, F182Y, and F182W showed products with a DP of up to 30, among which the most prominent products were those with a DP between 8 and 20 (30–50 min in Fig. 6*A*). In contrast, the F182A mutant accumulated products with a maximum DP of approximately 15, containing more 6-kestose-derived levan-type fructooligosaccharides compared with the WT. Similarly, the mutant Y237A produced products only up to decasaccharides and was the best FOS producer among the variants tested in this work, as it accumulated higher amounts of penta- to octasaccharides (11–28 min in Fig. 6*B*). On the other hand, N242A and K363A variants showed remarkable accumulation of small FOSs, up to tetra- and pentasaccharides, respectively. Among the small products, blastose, levanbiose, inulobiose, 1-kestose, 6-kestose, neo-kestose, 1,6-nystose, and 6,6-nystose could be identified according to standards previously reported (Fig. 6*C*) (20). It is worth mentioning that, for K363A, additional products with a DP of up to 9 were also detected but in very low amounts.

In terms of the hydrolysis/transfructosylation (H/T) partition, a decrease in transferability was observed in almost all the mutants (Table S2). Variant D117A had an H/T ratio similar to that of the WT (approximately 50/50), which was attributed to a noticeably higher accumulation of HMW levans. In contrast, mutants K363A and N242A preserved only 27% and 16% of transfructosylation, respectively, whereas the rest showed values between 40% and 47%.

Bearing in mind the enzyme modulation effect of the concentration enzyme on the SacB product specificity (10), these mutants were also assayed by employing a 10-fold higher enzyme concentration (1 μM, Fig. S3). This condition has an inhibitory effect on HMW levan synthesis in WT reactions, which leads to an almost exclusive production of LMW levans (MW 8.1 kDa). This effect was also observed in the mutant reactions, showing mainly LMW product distributions with average MWs of 7 (Y187A), 4.3 (F182A and D117A), and 3.4 (F182W), as well as the absence of polymeric products for mutants F182A, Y237A, K363A, and N242A (Fig. S4). Furthermore, oligosaccharide profiles similar to those under the 0.1 μM enzyme condition were obtained, showing only a slight increase in the product DP. Increases in the H/T ratio were also recorded for all variants compared with that of the WT and decreases in the H/T ratio were found for all variants compared with that under the 0.1 μM enzyme condition (Table S2).

## Discussion

This work presents, for the first time, the cocrystallization of an LS with a levan-type oligosaccharide. The levanhexaose observed in the OB1 site occupies the catalytic pocket from the bottom at subsite −1 and protrudes from the cavity toward the solvent. In this position, the oligosaccharide resembles a substrate prone to hydrolysis, considering the exolevanase activity reported for SacB (24); however, at the same time, the position might imply the conformation of a reaction product immediately after fructosyl transfer and before its release. In fact, the ligand employed corresponds to an intermediate product that is rapidly employed as acceptor substrate in nonprocessive levan synthesis from sucrose (20). Thus, this work described the positioning of a levan-type substrate/product inside the catalytic pocket filling the acceptor subsites +1, +2, +3, and +4 in an LS.

### Insights into the OB1 subsites +1 and +2

The SacB-levanhexaose complex showed no differences at subsite −1, which is highly specific for a fructosyl moiety and is supported by highly conserved residues in LSs and inulosucrases. Many of the residues in this site have been the subject of mutagenesis studies, generally resulting in mutants losing enzyme activity (22, 23, 25, 26, 27, 28, 29). However, at subsite +1, the SacB-levanhexaose complex highlights residues Y411 and K363 that interact with the ligand, and together with R360, which is an essential residue in the modulation of the SacB reaction specificity (hydrolysis/polymerization) (30), these residues seem to have coordinated flexible conformations depicted as different states in chains A and B. In chain A, the interactions might resemble an acceptor anchoring state, with R360 and K363 maintaining direct contact with fructosyl 2 of the ligand, while Y411 coordinates a water-mediated contact (Fig. S1). In contrast, chain B shows an open state, in which K363 no longer interacts with the ligand and whose position is now covered by R360. Meanwhile, Y411 approaches the ligand and now interacts directly with fructosyl 2. This rearrangement could be related to acceptor displacement into the catalytic cavity, as a movement necessary to allow the entry of a new donor molecule that reinitiates the transfer catalytic cycle.

Consequently, K363 might be an auxiliary residue in the binding of acceptor molecules at subsite +1 and participate in anchoring and releasing acceptor molecules. Accordingly, the mutation of K363 to alanine greatly impacted the enzymatic activity and eliminated the synthesis of LMW levans by limiting the efficient formation of oligosaccharides with a DP of up to 5, even though larger oligosaccharides were barely detected. A similar size-limiting effect was observed in alanine mutation studies targeting homologous residues in LSs from *B. megaterium* and *Bacillus licheniformis* RN-01 (12, 19), which are enzymes that natively accumulate LMW levans with DPs higher than 10 (31, 32). However, remarkably, K363A did not affect the formation of HMW levans by SacB, suggesting a nonessential role of this residue in processive levan synthesis.

Subsite +2 was previously identified and includes only residues involved in water-mediated interactions with the galactosyl moiety in the SacB-raffinose complex. In this work, we observed a water-mediated contact of K363 and direct contact with residues R246 and N242, suggesting their participation in the binding of acceptor substrates with a DP of 2 or higher. The auxiliary role of the side chain of K363 in subsite +2 correlates with the effect of the mutation of the homologous residue (K373) to arginine in *B. megaterium* LS, which limits the length of the oligosaccharides to nonasaccharides, in contrast to pentasaccharides obtained with K373A (12). R246 has, in fact, a relevant role in maintaining LS activity (22), which is most likely due to its participation in coordinating donor substrates at subsite −1. On the other hand, many reports have already highlighted the participation of N242 in the polymerization reaction of gram-positive LSs, and its mutation to alanine, histidine, tyrosine, or tryptophan invariably limited the products to tetrasaccharides (12, 17, 19, 33). Considering that the SacB mutant N242A also showed an increased *K*m, its deleterious effect may be related to the impaired coordination of sucrose as an acceptor molecule, thereby greatly affecting the formation of both HMW and LMW levans at the very beginning of the elongation process.

### Identification and implication of the OB1 site acceptor subsites +3 and +4 in the levan elongation mechanism

The SacB-levanhexaose complex establishes the existence of a subsite +3 that comprises residues D117, F182, and Y237, with the last participating through a water molecule. The stabilizer effect of the water-mediated interaction may be minor, considering that the mutation of Y247 (homologous to Y237) to phenylalanine preserved the kinetic and product profiles of *B. megaterium* LS (31). In contrast, the mutagenesis studies on D117 and F182 showed that these are essential residues participating in the elongation of levans, which most likely occurs through interactions with trisaccharides and larger acceptor molecules. Single substitutions of D117 with alanine and F182 with tyrosine or tryptophan must have disturbed the correct positioning of the acceptor molecules in this subsite, affecting the catalytic rate and limiting the final size of LMW products to 3.3 kDa (approximately half of the average size of WT LMW products), with no effect on the formation of HMW levans. Moreover, in the case of F182, the additional mutant F182A confirmed the role of this residue in the stabilization of W186, which is an amino acid involved in the binding of sucrose in subsites −1 and +1 (22, 23). Of interest, *B. megaterium* LS, which is characterized by accumulating mainly LMW levans with DPs of 2 to 20, contains a tyrosine as the residue homologous to F182 and an asparagine as the residue homologous to D117; therefore, according to our mutagenesis studies, these subtle changes may be responsible for the reduced size of the LMW products synthesized by this enzyme.

The last subsite identified (+4) includes only the residue Y237, which coordinates the binding of tetrasaccharides and larger substrates through a CH/π stacking mechanism. This hydrophobic interaction was previously hypothesized after observing that the substitution of this residue for other aromatic residues (phenylalanine or tryptophan) preserved the kinetic parameters and product profile of *B. megaterium* LS (12, 31). Conversely, mutations to alanine, isoleucine, and serine have been associated with the elimination of polymer formation and the synthesis of oligosaccharides consisting of up to 9 to 10 fructose units (12, 18). We evaluated the mutation Y237A in SacB and obtained the same oligosaccharide size limitation; however, we also observed that the synthesis of a HMW levan results in a reduced average molecular weight. Nevertheless, it is worth mentioning that the formation of HMW levan by Y237A was inhibited at high enzyme concentrations, which also occurred with the WT enzyme.

Finally, we studied residue Y187, which is located near Y237 but is beyond the sixth fructose unit of the levanhexaose molecule in the OB1 site. Once again, the mutation of Y187 to alanine did not affect the formation of HMW levans but slightly reduced the extension of the LMW levans, suggesting its possible participation in the last elongation stage of LMW products. In this regard, the homologous residue (Y197) in *B. megaterium* LS was mutated to phenylalanine, showing little change in enzyme specificity (31). In contrast, chemical derivatization of the enzyme in this position with a highly flexible tag that included a luminol derivative linked to an azido-1-deoxy-β-D-glucopyranoside surprisingly promoted the synthesis of HMW levans (31). Even though LSs from *B. subtilis* and *B. megaterium* have different product specificities, particularly regarding the extension of their LMW products (6.8 and 1.3 kDa, respectively), the region near the levanhexaose that protrudes outward into the SacB-levanhexaose complex seems to participate in levan elongation in both enzymes.

### Assembly *via* nonprocessive LMW levan synthesis in LSs

The SacB mutants defective in residues that interact with levanhexaose that were evaluated in this work showed an unfavorable effect on the ability to synthesize LMW levans, which are products associated with the nonprocessive elongation mechanism (10, 20). In fact, the ability to lengthen these products to a longer size was related to the external location of the mutated residues. Of interest, the mutants of residues belonging to the +2, +3, and +4 subsites retained the ability to produce HMW levans independently of the reduced size of the LMW products. In addition, this process could be inhibited by high enzyme concentrations, which is a key feature of SacB. Therefore, we can assume that the acceptor binding subsites +2, +3, and +4, as revealed by the SacB-oligosaccharide complex, correspond to the assembly line followed specifically during the nonprocessive synthesis of LMW levans, whereas the processive synthesis of HMW products could require the participation of different structural elements and, possibly, an additional pathway. Consequently, the acceptor interactions involved in the nonprocessive mechanism would be supported mainly by at least four binding subsites, whose strict integrity allows the synthesis of products with DPs between 2 and 70 in a MW distribution that averages 6.8 kDa. To understand the differences in the product size of the SacB mutants, we established a comparison with the mechanism proposed for the nonprocessive dextransucrase DSR-M from *Leuconostoc citreum* that considers the elongation process as a probability function that is increased when the chains reach a certain length defined by the acceptor subsites (34). Accordingly, for SacB mutants, the impaired binding subsites reduced the acceptor residence time in the catalytic pocket and its probability of elongation, leading to an increase in nonproductive encounters with the covalent fructosyl-enzyme intermediate and skewing the final distribution toward smaller levan chains.

**Figure 7:**
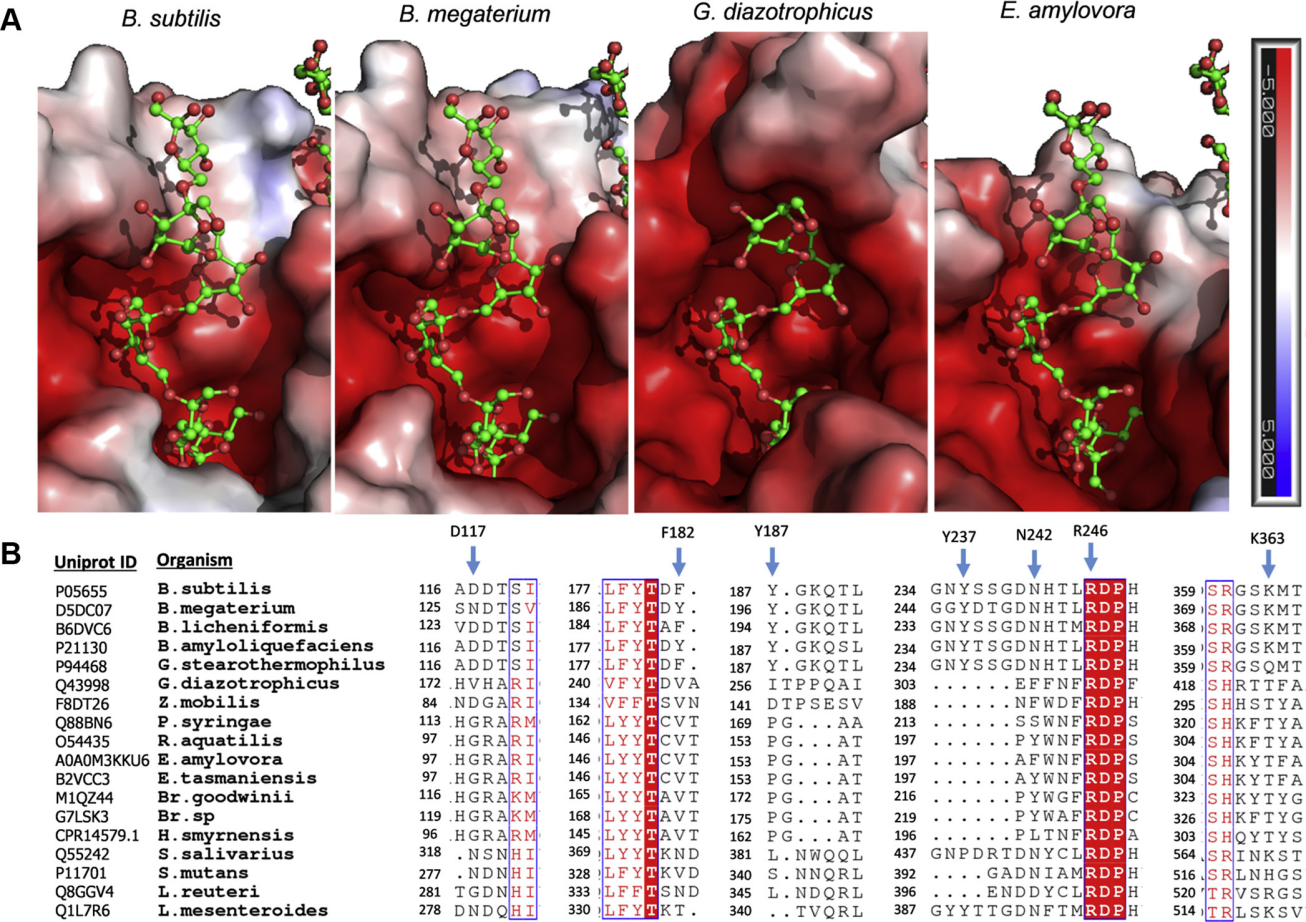
**Comparison of the OB1 site surface and sequence multialignment of SacB LS and its homologs.***A*, levanhexaose molecules (*green*) observed in the SacB–oligosaccharide complex (PDB ID: 6VHQ) were superimposed on the structures of three LSs: *B. megaterium* LS (PDB ID: 3OM2), *G. diazotrophicus* LS (PDB ID: 1W18), and *E. amylovora* LS (PDB ID: 4D47). The electrostatic potentials were calculated with Adaptive Poisson-Boltzmann Solver (APBS). *B*, sections of the multialignment were performed using Clustal Omega (54) and ESPript 3.0 (55). Amino acid residues that are strictly conserved are shown with a *red background*, and highly conserved residues are shown in *red font* and boxed in *blue*. Residues involved in subsites +2, +3, and +4 are indicated with *blue arrows*.

The nonprocessive synthesis of oligosaccharides is a phenomenon shared in the initial phase of the reaction used by all LSs. In LSs of gram-positive bacteria such as those from *Acetobacter diazotrophicus*, *Zymomonas mobilis*, *Halomonas smyrnensis*, *Pseudomonas syringae*, *E. amylovora*, and *E. tasmaniensis*, the nonprocessive reaction leads to the accumulation of mainly short-chain FOSs (35, 36, 37, 38, 39). In contrast, larger oligosaccharides forming a distribution of LMW levans (1.6–8 kDa) have been reported mainly in LSs of bacteria in the genus *Bacillus*, such as the LSs of *B. subtilis* (strains 168, Natto and C4), *B. megaterium*, and *B. licheniformis* RN-01 (12, 32, 40, 41, 42). The different product specificities between LSs of different origins can be related to differences in the enzyme surface topology related to the conservation degree of the residues belonging to the binding subsites +2, +3, and +4 (Fig. 7, *A* and *B*). Except for R246, which is conserved in all LSs, all other residues of these subsites are conserved in LSs of the genus *Bacillus* (Fig. 7*A*). By comparing the OB1 site surfaces, we can see that the *B. megaterium* LS shares a topology capable of binding the levanhexaose molecule through the same binding subsites. In contrast, the LSs of *G. diazotrophicus* and *E. amylovora* show different topologies, especially in the +3 and upper subsites. The *G. diazotrophicus* LS has a protuberance, whereas the *E. amylovora* LS has a valley, both of which hinder the positioning of levanhexaose. This comparison suggests that the topology of this area is responsible for allowing the elongation of acceptors toward LMW levans in *B. subtilis* and *B. megaterium* LSs, which obstructs the elongation of acceptors in the case of *G. diazotrophicus* LS and does not support the elongation of acceptors in the case of the *E. amylovora* LS, yielding only small FOSs in these cases.

### OB2 site possible implications in the recognition of acceptor substrates and levan polymerization

Owing to the structure of the SacB-levanhexaose complex, a second OB site in close vicinity to the catalytic cavity was revealed for the first time in *B. subtilis* levansucrase. This secondary site supported the binding of a levanhexaose molecule mainly by lodging two of its fructosyl moieties. Similarly, a secondary OB site distant from the catalytic site was recently identified in the catalytic pocket periphery of *E. tasmaniensis* LS, in which levanbiose was observed (14). This disaccharide is located at 22.5 and 24.8 Å over the surface from the hexasaccharides bound to the OB1 and OB2 sites observed in the SacB–oligosaccharide complex (Fig. S5). The authors suggest that the presence of the levanbiose-binding OB2 in the enzyme surface might promote contacts between neighboring enzyme molecules in solution, thereby increasing the probability of enzyme–product interactions in the active site. In transglycosidases and glycosidases, secondary OB sites have also been reported to perform functions such as guiding the substrate into the active site, enhancing processivity and regulating allosteric sites (43). Accordingly, a secondary OB subsite of SacB may support the levan elongation mechanism by retaining the fructose acceptor and preventing its disassociation from the enzyme, thus increasing the processivity and polymer production. Given the proximity and parallel positioning of both ligands in the OB1 and OB2 sites, as both are located with the reducing end pointing toward the solvent, the role of the OB2 site may be related to the elongation of branches during levan synthesis. This kind of auxiliary role has been proposed as the secondary OB site identified in amylosucrase from *Neisseria polysaccharea*, which serves as an anchoring platform to capture a glycogen branch at the enzyme surface while other branches are elongated in the active site through incoming and outgoing movements. In this way, the secondary OB site can support a so-called semiprocessive mechanism (44).

**Figure 8:**
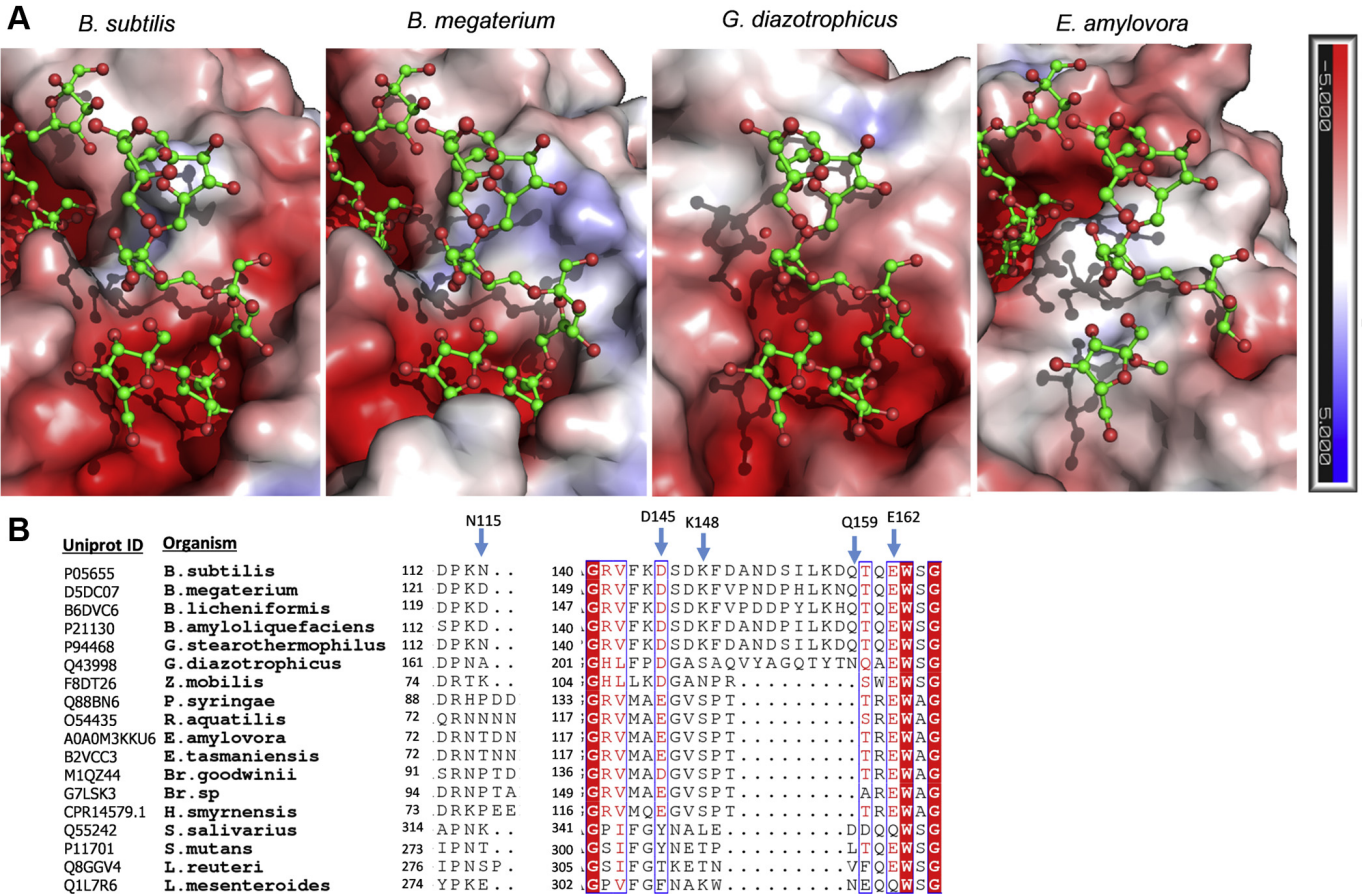
**Comparison of the OB2 site surface and sequence multialignment of SacB LS and its homologs.***A*, levanhexaose molecules (*green*) observed in the SacB–oligosaccharide complex (PDB ID: 6VHQ) were superimposed on the structures of three LSs: *B. megaterium* LS (PDB ID: 3OM2), *G. diazotrophicus* LS (PDB ID: 1W18), and *E. amylovora* LS (PDB ID: 4D47). The electrostatic potentials were calculated with Adaptive Poisson-Boltzmann Solver (APBS). *B*, sections of the multialignment were performed using Clustal Omega (54) and ESPript 3.0 (55). Amino acid residues strictly conserved are shown with a *red background*, and highly conserved residues are shown in *red type* and boxed in *blue*. Residues involved in the OB2 site are indicated with *blue arrows*.

In the OB2 site, five residues stabilize the levanhexaose molecule through direct interactions, namely, N115, D145, K148, Q159, and E162. Most of these residues are conserved in enzymes from gram-positive bacteria, particularly in the members of the Bacillaceae family (Fig. 8, *A* and *B*), and only the functional role of the E162 residue, which was conserved in almost all enzymes compared in this study, has been determined in *Z. mobilis* LS (homologous to E117). Its substitution for glutamine decreased the *K*m by four times, increased the transfructosylation activity by 25% and favored polymer formation (45). Accordingly, it is possible that the other residues belonging to the OB2 site participate in the activity and polymerization reaction in SacB. Topological comparison of the OB2 sites of SacB and other LSs showed a negatively charged solvent-exposed groove in the LSs from *B. subtilis*, *B. megaterium*, and *G. diazotrophicus*, suggesting that these LSs could support the binding of an oligosaccharide in this area (Fig. 8*A*). The planar, noncharged surface of the *E. amylovora* LS probably could not support this interaction. In addition, it is worth noting that there is a channel connecting the OB1 and OB2 sites in the *B. subtilis* and *B. megaterium* structures, which lodges a bromide molecule in both chains A and B of the SacB-levanhexaose complex. It is feasible that the residues present in this channel may participate as stabilizers of the acceptor molecule; however, studies are necessary to support this hypothesis.

## Conclusions

The elucidation of the SacB structure in complex with a levan-type hexasaccharide allowed us to identify, for the first time, the accurate placement of a levan substrate/product inside and beyond the central catalytic cavity, as well as to identify the residues belonging to the subsites responsible for this arrangement. The SacB-oligosaccharide interaction is supported by at least four acceptor-binding subsites that integrate the assembly line employed during the nonprocessive synthesis of LMW levans. The structural comparison of different LSs suggests that the topological differences in the identified upper acceptor subsites are responsible for the distinct product specificities of the Bacillaceae family and gram-positive LSs regarding the size of LMW products, including short FOSs and levans. Our results also support the idea of an additional pathway during the processive synthesis of HMW levans that remains to be clarified.

In addition, a secondary OB site in *B. subtilis* LS in the vicinity of the catalytic pocket was also revealed for the first time. Given its proximity, this site may participate in the elongation of larger products or be related to the formation of branches during levan synthesis. The precise function of the ancillary site is currently under investigation.

## Experimental procedures

### Cocrystallization and crystal structure determination

The oligosaccharide 6,6,6,6,6,6-kestooctaose was isolated from a 50 ml SacB reaction (1 μM enzyme and 600 g/l sucrose, pH 6, 37 °C) by size exclusion chromatography followed by reverse phase HPLC. Size exclusion chromatography fractionation was performed in a Bio-Gel P2 Extra Fine column (2.5 × 100 cm, Bio-Rad) using deionized water as eluent at 0.2 ml/min, and reverse phase HPLC was carried out in a Waters 1525 HPLC system equipped with a Spherisorb S5 ODS2 Semi-Prep column (20 × 250 mm, Waters), employing deionized water as the mobile phase at 7 ml/min. Crystals of an inactive double-mutant enzyme SacB (D86A/E342A) were obtained by cocrystallization with 6,6,6,6,6,6-kestooctaose using microbatch method at 18 °C (46). The enzyme and ligand concentrations were 32.5 and 12.5 mg/ml (molar ratio of 15), respectively. The drops were prepared manually in 72-well crystallization plates from Greiner, Hampton Research (Aliso Viejo, CA, USA) by mixing the enzyme/octosaccharide (1.0 μl) (in 100 mM 2-(N-Morpholino)ethanesulfonic buffer, pH 6.0) with the crystallization solution (1.0 μl) containing 30% w/v PEG 2000 monomethyl ether and 150 mM potassium bromide. The drops were covered with paraffin oil (10 μl) (46). Suitable crystals for diffraction appeared after 1 month and continued to grow for about 1 month. Crystals for data collection were then flash cooled by immersion in liquid nitrogen using paraffin oil as cryoprotectant.

Diffraction data of one crystal were collected on a rotating anode diffractometer (Rigaku RU200H) of the “Laboratorio Nacional de Estructura de Macromoléculas” (LANEM, Instituto de Química, UNAM), using a Pilatus 200K detector. A diffraction data set was indexed, integrated, and scaled with the program HKL-3000 (47). Crystal belongs to primitive monoclinic space group P2_1_ with cell dimensions *a* = 69.3 Å, *b* =78.6 Å, *c* = 78.7 Å, and *β* = 93.9° (Table S1).

The structure was determined by molecular replacement using the available coordinates of levansucrase of *B. subtilis* (PDB ID: 1OYG) as the starting model (8). The search was performed with the program Phaser (48). The models were improved by rigid body refinement and geometric constraint with REFMAC (49). Afterward, refinement was performed by alternating cycles of automatic and manual refinement with PHENIX (50) and COOT (51), respectively. Molecules of fructose were modeled with COOT (51) and its geometry minimization with the program eLBOW of PHENIX (50) and manually fitted into the electron density map. The crystal structure has a final R of 16.7% (R_free_ of 23.4%, calculated with 5% of the data randomly selected) at 2.05 Å resolution (Table S1). The final structure displayed good stereochemistry as analyzed by MolProbity (52).

### Site-directed mutagenesis

The selected mutations were introduced into the levansucrase gene (SacB) of *B. subtilis* strain 168 cloned into the pET22b vector, using the QuickChange II Site-Directed Mutagenesis (Stratagene, USA) kit according to supplier specifications. The mutant Y187A was purchased from Mutagenex (USA). The sequences of the oligonucleotides used for mutagenesis are shown in the Table S3. The constructs obtained were verified in an automatic DNA sequencer model 3130xl (Applied Biosystems, Thermo Scientific) at the DNA Synthesis and Sequencing Unit of the Institute of Biotechnology (UNAM).

### Expression and purification or LS variants

Fresh *Escherichia coli* BL21 (DE3) transformants bearing selected plasmid were used to inoculate 50 ml LB medium containing 200 μg ml^−1^ ampicillin and incubated over night at 37 °C. Precultures were used to inoculate 1 l LB medium with 200 μg ml^−1^ ampicillin and incubated at 37 °C and 200 rpm until an optical density of 0.6 A_600nm_ was reached. Expression of levansucrase was induced by adding IPTG at a final concentration of 0.2 mM during 8 h at 18 °C and 120 rpm. Cells were harvested by centrifugation and resuspended in 100 mM sodium acetate buffer pH 6.0. Cell lysis was performed through lysozyme treatment, three freeze-thawing cycles, and sonication. After extract centrifugation, the cleared lysate was loaded onto a 5-ml HiTrap CM-sepharose Fast Flow column (GE Healthcare, USA) and levansucrase eluted with a 1 M sodium acetate buffer pH 6.0 in an AKTA prime system (Amersham Pharmacia Biotech, UK). The eluted protein fractions were pooled, concentrated, and buffer exchanged to 50 mM sodium acetate buffer pH 6.0 added with 1 mM CaCl_2_ (buffer also employed in reactions), using Amicon Ultra-4 filters with a 30 kDa molecular weight cutoff (Merck Millipore, Germany). The colorimetric Bio-Rad Protein Assay was used to determinate the concentration of purified enzymes using bovine serum albumin (Bio-Rad) as the standard.

### Determination of kinetic parameters

Catalytic parameters of levansucrase variants were obtained from reactions carried out in 50 mM acetate buffer pH 6.0 containing varying concentrations of sucrose (0–300 mM) and purified variant. Initial rates were determined by measuring the release of reducing sugars over the time course with the dinitrosalicylic acid method (53). *K*m and *V*max values were calculated by nonlinear fitting to the Michaelis–Menten equation using OriginPro (OriginLab, USA). *K*_cat_ was calculated considering the enzyme molecular weight (52.3 kDa). Experiments were performed in duplicate.

### Product analyses

The product specificity of SacB and its variants was determined by carrying out 600-μl reactions containing 50 mM acetate buffer pH 6.0, 300 mM sucrose, and 0.1 μM (5.5 μg/ml) or 1 μM (55 μg/ml) of each purified enzyme. Enzymatic reactions were performed at a defined enzyme concentration instead of enzyme activity bearing in mind the enzyme concentration effect on size product previously reported (10). Reactions were let to proceed at 37 °C and 350 rpm until high sucrose conversion was reached. The necessary time for each reaction was estimated considering the enzyme concentration and *k*_cat_ of the variant. Reactions were stopped by heating in boiling water for 10 min and frozen until analysis. All experiments were performed at least in duplicate.

Simple sugars (fructose, glucose, sucrose) were quantified by HPAEC-PAD in a Dionex ICS-5000 DP system (Thermo Scientific, USA) utilizing CarboPac PA1 columns (guard, 4 × 50 mm; main column, 4 × 250 mm) with a gold working electrode and Ag/AgCl reference electrode. Isocratic elution was performed using 200 mM NaOH at 1 ml min^−1^ for 8 min followed by washing (8 min using 200 mM NaOH + 500 mM) and re-equilibration (8 min at initial conditions) steps. The H/T ratio was obtained from the values of free fructose (F) and glucose (G) according to the following equations:

$$\%H=\frac{F}{G}$$

$$\%T=\frac{G-F}{G}$$

Oligosaccharide analysis was performed by HPAEC-PAD using CarboPac PA-200 columns (guard, 3 × 50 mm; main column, 3 × 250 mm) maintained at 30 °C. Oligosaccharide separation was obtained applying two linear sodium acetate gradients with 100 mM NaOH at 0.5 ml min^−1^ as follows: 5 to 100 mM sodium acetate in 25 min, 100 to 400 mM in 60 min, and 10 min for initial condition re-equilibration (5 mM sodium acetate). The product identification was achieved by comparison with standards previously reported (20).

Polymer molecular weight analysis was carried out by gel permeation chromatography using two Waters Ultrahydrogel columns in series (Ultrahydrogel Linear, 7.8 × 300 mm; Ultrahydrogel 500, 7.8 × 300 mm) maintained at 30 °C, in a Waters Alliance 2695 system (Waters, USA) coupled with a Waters 2414 refractive index detector (Waters, USA). Isocratic elution was developed using 0.1 M NaNO_3_ as the mobile phase at a flow rate of 0.8 ml min^−1^. The molecular mass was determined according to a calibration curve constructed with dextran standards (Sigma-Aldrich, USA).

### Computational analyses

Multiple sequence alignment of levansucrases was generated using Clustal Omega (54), and the results were rendered using ESPrit 3.0 (55). Structural alignments, RMSD calculations, and model figures were performed using the PyMOL Molecular Graphics System, Version 1.8 (Schrödinger, USA).

## Data availability

Coordinates and the structural factors of SacB-levanhexaose complex have been deposited in the Protein Data Bank (http://www.wwpdb.org/) under code 6VHQ.

## Conflict of interest

The authors declare that they have no conflicts of interest with the contents of this article.
