# Supplementary material for: The molecular basis of the nonprocessive elongation mechanism in levansucrases
Source: J Biol Chem. 2020 Dec 17;296:100178. doi: 10.1074/jbc.RA120.015853 (PMC7948499; doi:10.1074/jbc.RA120.015853)
Supplement: Supplementary Figures and Tables [file mmc1.pdf]

The molecular basis of the nonprocessive elongation mechanism in levansucrases

Enrique Raga-Carbajal[1‡] Adelaida Díaz-Vilchis[2‡], Sonia P. Rojas-Trejo[2], Enrique Rudiño-Piñera[2] and Clarita Olvera[1*]

From [1]Departamento de Ingeniería Celular y Biocatálisis, Instituto de Biotecnología, Universidad Nacional Autónoma de México, Av. Universidad 2001, Col. Chamilpa, 62210 Cuernavaca, Morelos, México. [2]Departamento de Medicina Molecular y Bioprocesos, Instituto de Biotecnología, Universidad Nacional Autónoma de México, Av. Universidad 2001, Col. Chamilpa, 62210 Cuernavaca Morelos, México.

Running title: *Crystal structure of the SacB-oligosaccharide complex*

*To whom correspondence should be addressed: Departamento de Ingeniería Celular y Biocatálisis, Instituto de Biotecnología, Universidad Nacional Autónoma de México, Av. Universidad 2001, Col. Chamilpa, 62210 Cuernavaca, Morelos, México; clarita@ibt.unam.mx; Tel (52) 7773291600 Ext. 38117.

‡Both authors contributed equally to this work.

Supporting Information

Table S1. X-ray Data Collection and Refinement Statistics of SacB-Oligosaccharide Complexes

PDB ID	6VHQ
Data collection	
Wavelength (Å)	1.54
Space group	$P2_1$
Cell dimensions	
a (Å)	69.3
b (Å)	78.6
c (Å)	78.7
β (°)	93.9
Resolution (Å)	25.0 - 2.05 (2.09 - 2.05)
Unique reflections	53 175 (5145)
$I/\sigma I$	23.9 (3.5)
R_{merge} (%)	4.8 (52.6)
$CC_{1/2}$	0.98 (0.82)
Completeness (%)	99.9 (100.0)
Multiplicity	2.7 (2.5)
Mosaicity (°)	0.65
Refinement	
Resolution (Å)	25.0 - 2.05
R (%)	16.7
R_{free} (%)	23.4
Number of atoms	
Protein	7 097
Calcium	2
Bromide	9
Fructose	223
Water	540
B-factors (Å^2)	
Protein	27.3
Calcium	15.0
Bromide	37.2
Fructose	47.2
Water	33.5
All atoms	28.3
Wilson Plot	25.1
RMSD	
Bond lengths (Å)	0.016
Bond angles (°)	1.48
Ramachandran plot	
Most favoured regions (%)	96.0
Additional allowed regions (%)	3.5
Disallowed regions (%)	0.34

Statistics for the highest-resolution shell are shown in parentheses.

Table S2. Hydrolysis and transfructosylation values obtained in reactions with SacB and its mutants.

	0.1 µM		1 µM	
	Transfructosylation (%)	Hydrolysis (%)	Transfructosylation (%)	Hydrolysis (%)
**WT**	52.1 ± 1.0	47.9 ± 1.0	41.7 ± 1.3	58.3 ± 1.3
**Y187A**	47.6 ± 2.3	52.4 ± 1.0	35.1 ± 0.1	64.9 ± 0.1
**Y237A**	40.7 ± 0.7	59.3 ± 0.7	30.1 ± 1.2	69.9 ± 1.2
**D117A**	50.6 ± 1.3	49.4 ± 1.3	33.1 ± 0.2	66.9 ± 0.2
**F182Y**	44.8 ± 3.2	55.2 ± 3.2	24.2 ± 1.8	75.8 ± 1.8
**F182W**	43.2 ± 1.3	56.8 ± 1.3	30.0 ± 0.3	70.0 ± 0.3
**F182A**	42.0 ± 0.4	58.0 ± 0.4	18.9 ± 3.0	81.1 ± 3.0
**K363A**	27.4 ± 1.7	72.6 ± 1.7	20.2 ± 1.3	79.8 ± 1.3
**N242A**	16.0 ± 0.9	84.0 ± 0.9	19.0 ± 1.8	81.0 ± 1.8

Table S3. Oligonucleotides used for site-directed mutagenesis.

Oligonucleotide	DNA sequence (5' to 3')
N242A Fw	C TAC AGC TCA GGC GAC GCG CAT ACG CTG AGA GAT CC
N242A Rv	GG ATC TCT CAG CGT ATG CGC GTC GCC TGA GCT GTA G
K363A Fw	CT GAC TCC CGC GGA TCA GCG ATG ACG ATT GAC GGC
K363A Rv	GCC GTC AAT CGT CAT CGC TGA TCC GCG GGA GTC AG
Y237A Fw	C GAT GAA GGC AAC GCG AGC TCAGGC GAC AAC CAT ACG
Y237A Rv	CGT ATG GTT GTC GCC TGA GCT CGC GTT GCC TTC ATC G
F182A Fw	CGT TTA TTC TAC ACT GAT GCG TCC GGT AAA CAT TAC GGC
F182A Rv	GCC GTA ATG TTT ACC GGA CGC ATC AGT GTA GAA TAA ACG
F182W Fw	CGT TTA TTC TAC ACT GAT TGG TCC GGT AAA CAT TAC GGC
F182W Rv	GCC GTA ATG TTT ACC GGA CCA ATC AGT GTA GAA TAA ACG
F182Y Fw	CGT TTA TTC TAC ACT GAT TAT TCC GGT AAA CAT TAC GGC
F182Y Rv	GCC GTA ATG TTT ACC GGA ATA ATC AGT GTA GAA TAA ACG
D117A Fw	GGA GAT CCT AAA AAT GCG GCG GAC ACA TCG ATT TAC ATG
D117A Rv	CAT GTA AAT CGA TGT GTC CGC CGC ATT TTT AGG ATC TCC
D145A Fw	CT GGC CGC GTC TTT AAA GCG AGC GAC AAA TTC GAT GC
D145A Rv	GC ATC GAA TTT GTC GCT CGC TTT AAA GAC GCG GCC AG
Y187A	GGT AAA CAT GCG GGC AAA CAA

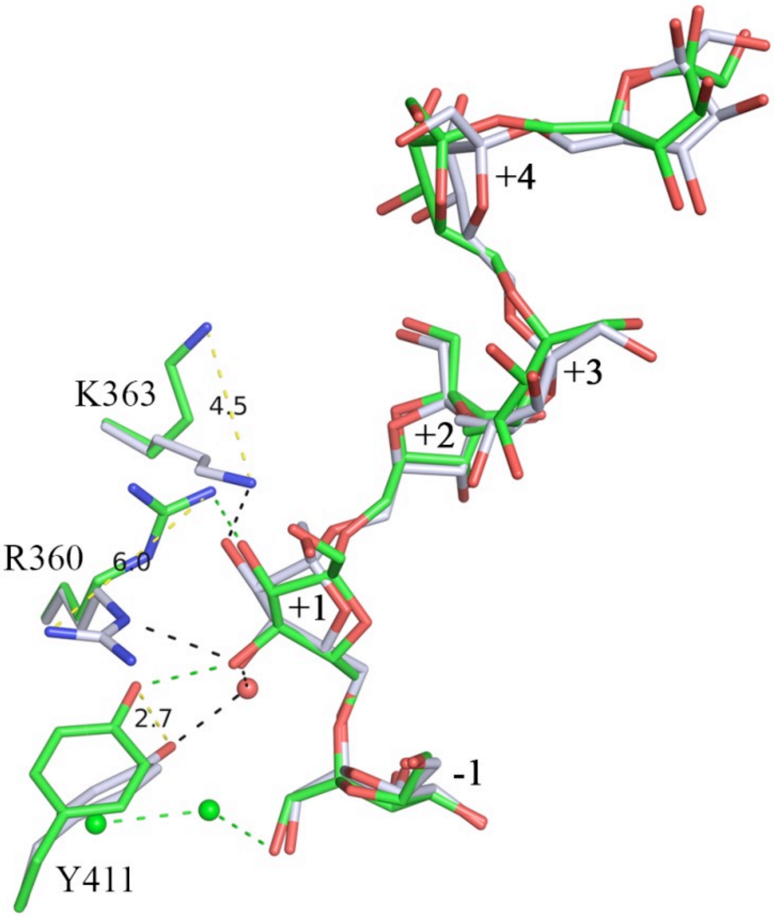

Figure S1. Structural comparison of R360, K363, Y411 and levanhexaose between chains A (grey) and B (green) of the SacB-levanhexaose active site. Hydrogen bonds are shown as black and green dashed lines for chains A and B, respectively. Distances are indicated for different conformations between both chains.

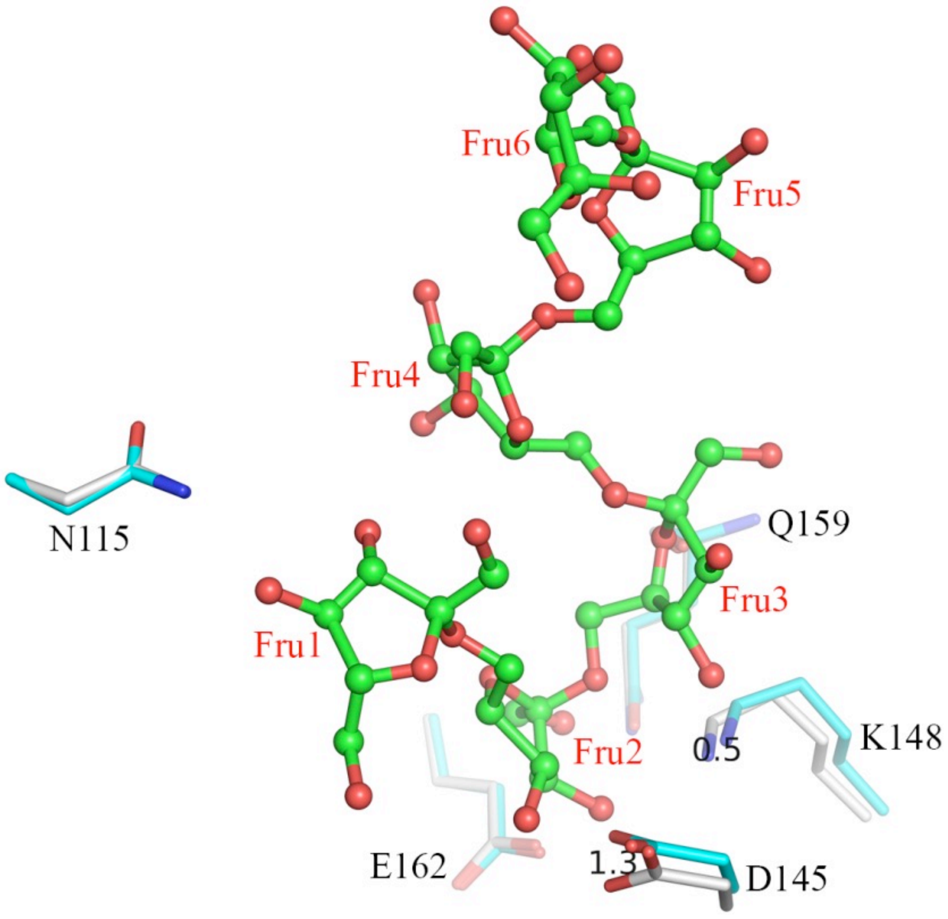

Figure S2. Superimposed residues at the OB2 of SacB (D86A/E342A)-levanhexaose and the SacB apo form. SacB-levanhexaose (gray, PDB ID: 6VHQ) and SacB Apo (cyan, PDB ID: 1OYG). Distances are indicated for different conformations between the ligand and apo forms.

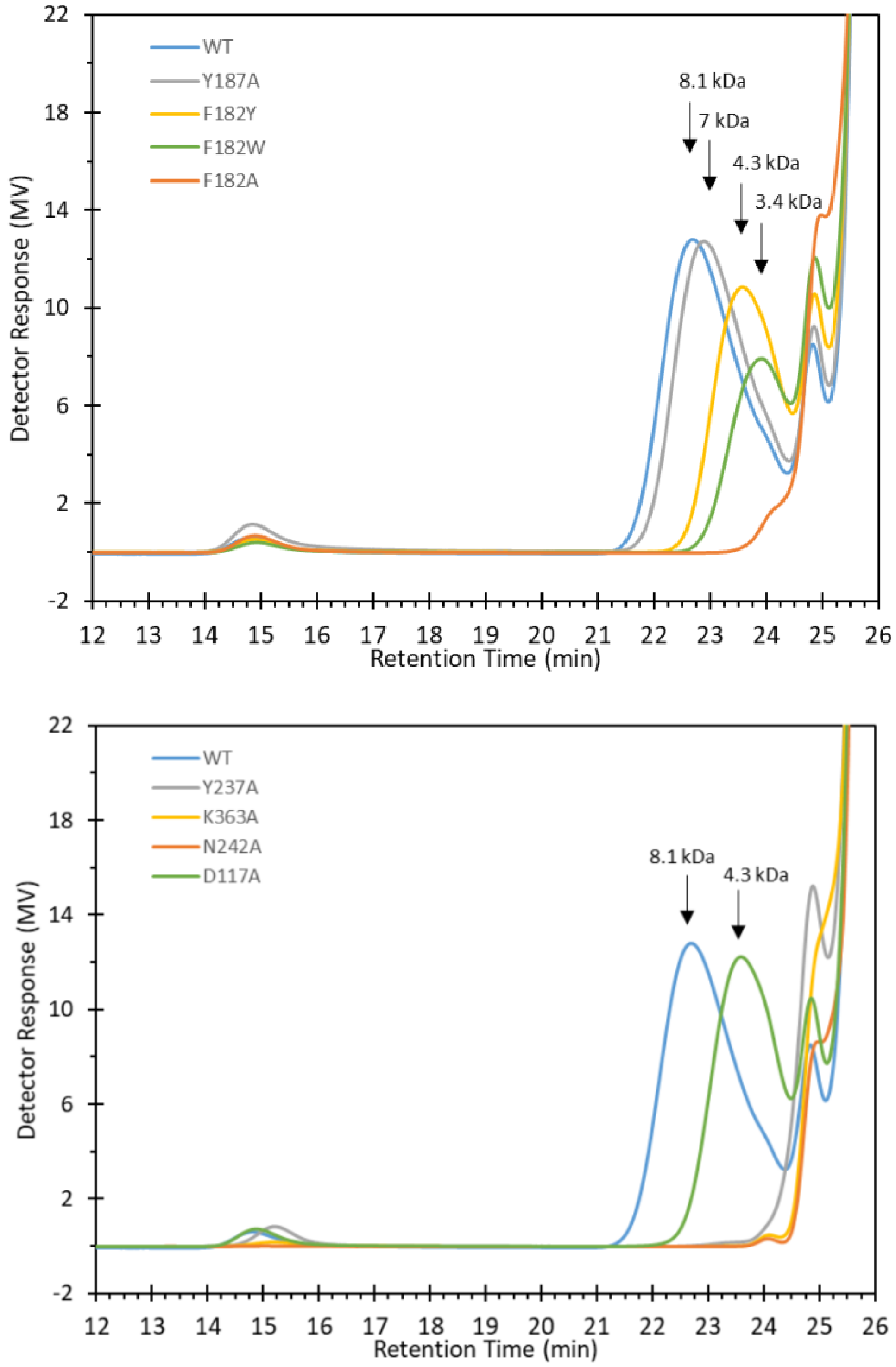

Figure S3. Gel permeation chromatograms of the products synthesized by SacB and its mutants. Reaction conditions: 1 µM enzyme, 300 mM sucrose, 37 °C, pH 6. Reactions were analyzed at a sucrose conversion of over 95%.

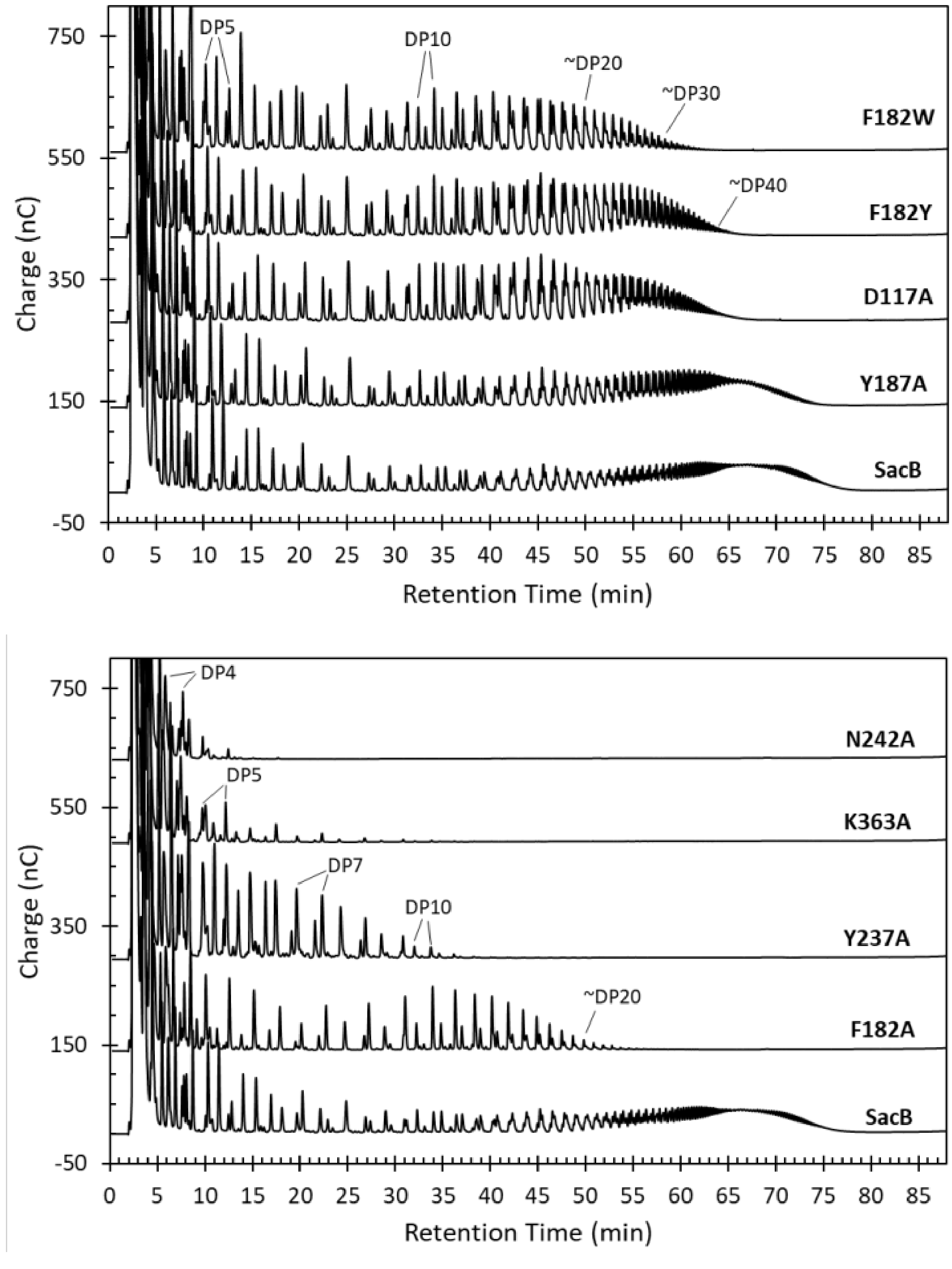

Figure S4. HPAEC-PAD analysis of the oligosaccharides synthesized by SacB and its mutants. Reaction conditions: 1 µM enzyme, 300 mM sucrose, 37 °C, pH 6. Reactions were analyzed at a sucrose conversion of over 95% (A and B).

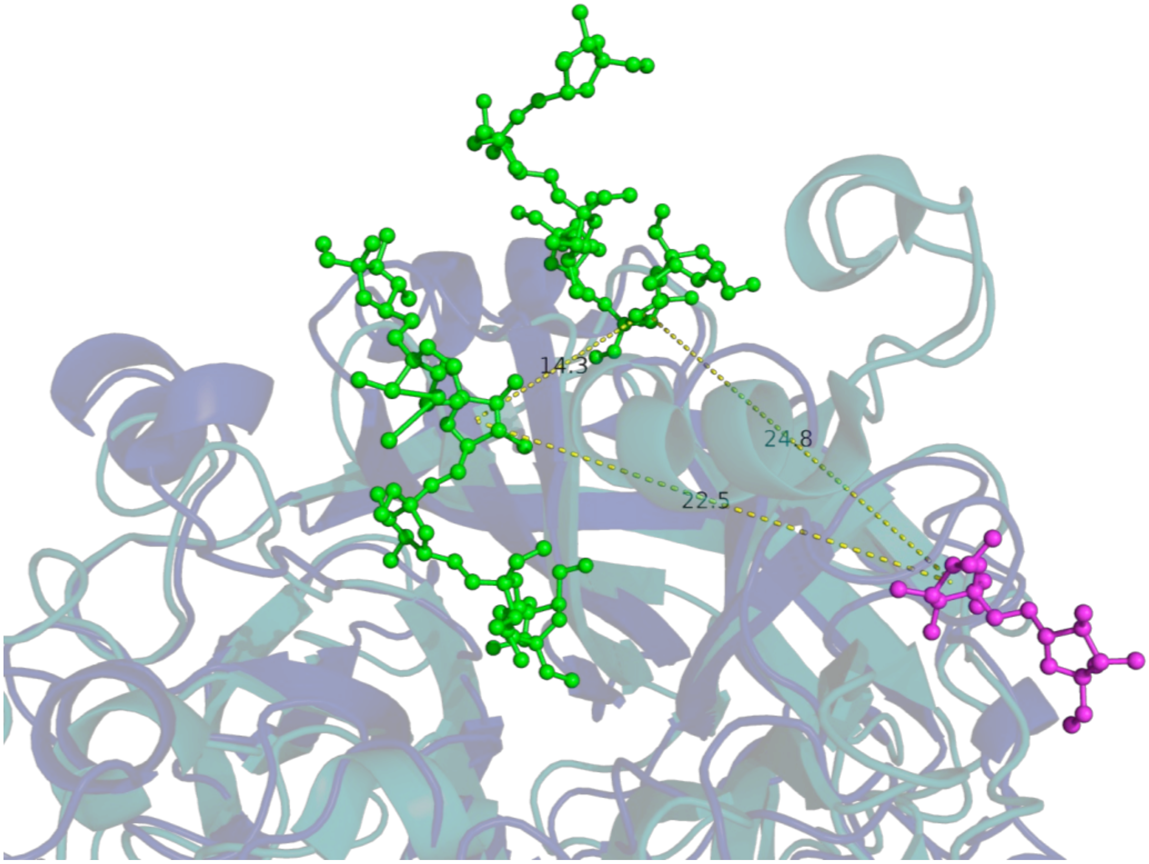

Figure S5. Superimposition of the levanhexaose-SacB complex (blue, PDB: 6VHQ) and *E. tasmaniensis* LS-levanbiose complex (cyan, PDB: 6RV5). The measured distances between levanhexaose molecules (green) and levanbiose (magenta) are indicated as yellow dashed lines.
